# Developing Academic–Practice Partnerships to Enhance the Integration of Genomics Into Public Health

**Published:** 2005-03-15

**Authors:** Karen L Edwards, Sarah F Raup, Kristin Peterson Oehlke

**Affiliations:** Department of Epidemiology; The author is also affiliated with the Institute for Public Health Genetics, School of Public Health and Community Medicine, University of Washington, Seattle, Wash; Department of Epidemiology, School of Public Health and Community Medicine, University of Washington, Seattle, Wash; Minnesota Department of Health, Minneapolis, Minn

## Abstract

The sequencing of the human genome has provided tools to gain a better understanding of the role of genes and their interaction with environmental factors in the development of disease. However, much work remains in translating discoveries into new opportunities for disease prevention and health promotion. Both public health academia and practice have important roles to play in bridging the gap between the growth in knowledge stemming from the Human Genome Project and its application in public health. Recognizing this, the Centers for Disease Control and Prevention, through the Association of Schools of Public Health, established Centers for Genomics and Public Health at three schools of public health in 2001: the University of Michigan, the University of North Carolina, and the University of Washington. This paper describes the experience of the University of Washington Center for Genomics and Public Health in forging partnerships with public health practitioners to translate genomic advances into public health practice.

## Introduction

The sequencing of the human genome has provided tools to gain a better understanding of the role of genes and their interaction with environmental factors in disease development (1). This understanding is predicted to improve methods for targeting interventions aimed at preventing disease and improving health. In the future, for instance, some believe that individuals will be screened for genetic susceptibility to common disorders such as cancer, heart disease, and diabetes, thus yielding recommendations for personalized prevention strategies. Primary prevention strategies (such as dietary changes) and secondary prevention strategies (such as more frequent or earlier initiation of medical screening) might be used to minimize disease risk (2). Disease management is expected to improve through pharmacogenomics, which promises safer and more efficacious drugs through the customization of drug therapies based upon an individual's genetic makeup (3).

Much work remains, however, in translating discoveries into new opportunities for disease prevention and health promotion. While new reports of gene–disease associations are published almost daily, most are not replicable (4). Most chronic diseases are caused not by a single gene but by the complex interplay among several genes and numerous environmental factors. Therefore, population-based studies that assess the prevalence of genotypes, the disease risk associated with gene variants, and gene–gene and gene–environment interactions are needed (5). Furthermore, as new genetic tests are developed, information on their analytical validity (accuracy with which a genetic characteristic can be detected in a given laboratory test), clinical validity (accuracy with which a test predicts a clinical outcome), clinical utility (likelihood that the test will lead to an improved health outcome), and ethical, legal, and social consequences will be needed to make decisions about their use in clinical and public health practice (6). The impact of genomic information on risk perception and health behavior change needs to be better studied, and the added value of targeted interventions based upon genetic susceptibility compared with population-based prevention recommendations needs to be determined (7). Health care providers and public health professionals will need to be educated about genomics (7) and the public will need to be "genetically literate" if genomics is to be used as a tool for disease prevention (8). Concerns about the use of genomic information (e.g., fear of employment or insurance discrimination) will need to be well understood through community input and adequately addressed as programs or policies incorporating genomics are developed (9).

## The Centers for Genomics and Public Health: Linking Academia and Practice

Both public health academia and practice have important roles to play in bridging the gap between the growth in knowledge stemming from the Human Genome Project and its application in improving health and preventing disease. Recognizing this, the Centers for Disease Control and Prevention's Office of Genomics and Disease Prevention (CDC OGDP), through the Association of Schools of Public Health, established Centers for Genomics and Public Health at three schools of public health in 2001: the University of Michigan, the University of North Carolina, and the University of Washington. CDC OGDP, which since 1997 has taken the lead in promoting the use of genomics to improve health and prevent disease across the lifespan by integrating genomics into public health research, policy, and programs (10), established the centers with the mission of further integrating genomics into public health practice by increasing the genomics and public health knowledge base; providing technical assistance to local, state, and regional public health systems; and training the public health workforce (11). In 2002, the Institute of Medicine released the report *Who Will Keep the Public Healthy? Educating Public Health Professionals for the 21^st^ Century*. The report acknowledged genomics as an important component of public health and called upon schools of public health to provide students and practicing public health professionals with a framework for understanding the importance of genomics to public health (12). Although the centers were established prior to development of this report, they are clearly playing a role in answering this call.

To accomplish their mission of further integrating genomics into public health practice, particularly in the area of chronic disease prevention, the centers have developed strong ties with public health practitioners within state public health agencies and to a lesser extent with practitioners at the local level. Both academics and practitioners have benefited from partnerships through new concepts and applications. For instance, though some researchers within schools of public health have long been engaged in a variety of research activities related to genomics in the areas of biostatistics, epidemiology, environmental and occupational health, health policy, health services, and the behavioral sciences, these researchers rarely engage in public health activities at the state or local level, and many do not have a solid understanding of public health in the real world. State health departments have also been engaged in genomics activities for a number of years, ranging from newborn screening programs to the provision of genetic services. However, a 2000 survey of state health departments conducted by the Council of State and Territorial Epidemiologists indicated that few state health departments had begun to consider opportunities for using genomics outside of the context of maternal and child health, despite an increasing awareness of the potential application of genomics in broader public health efforts. Survey respondents identified lack of resources, proven disease prevention measures, and outcomes data as potential barriers (13). Through the centers, academic researchers and public health practitioners have begun to collaborate more closely, and opportunities for using genomics to improve public health, particularly in the area of chronic disease prevention, have been identified as a result. This paper describes the experience of the University of Washington Center for Genomics and Public Health (UWCGPH) in forging partnerships with public health practitioners to translate genomic advances into public health practice.

## The University of Washington Center for Genomics and Public Health

At UWCGPH, we have learned a tremendous amount about how to develop genomics-centered collaborations with state public health agencies, and these collaborations enhance the work of both researchers and practitioners. Relationship building is a first step toward identifying opportunities for collaboration. Despite the distance that may lie between universities and state health agencies, face-to-face contact and regular communication is important to developing relationships. We traveled from Seattle to the Washington State Health Department (WA DOH) in Olympia, Wash, numerous times to meet with chronic disease program staff. We also traveled to the Oregon Health Division (OHD) in Portland to meet with genetics program staff; Oregon is one of four states currently leading efforts to integrate genomics into chronic disease with recent funding from the CDC's National Center for Chronic Disease Prevention and Health Promotion (14). We attended several public health conferences and participated in evaluating state public health programs to learn more about public health activities in Washington. All these efforts provided us with the opportunity to better understand the role of state public health programs and the knowledge and skills held by public health practitioners, as well as to share information about our expertise and to work together to identify ways in which genomics might be integrated into state public health efforts.

We have found that both formal and informal educational efforts are effective for stimulating interest in genomics and encouraging ongoing learning about genomics terms and concepts. For example, we collaborated with the CDC and other Centers to develop an animated Web-based module, *Genomics for Public Health Practitioners: The Practical Application of Genomics in Public Health Practice*. Pilot testing of this module indicates that it addresses many questions raised by public health practitioners about the use of genomics in public health. We also identified genomics training courses within the Pacific Northwest for interested public health practitioners to attend. While such formal educational efforts are effective in some instances, we also believe that adding genomics terms and concepts to the public health lexicon can be accomplished through ongoing joint efforts between the centers and state health departments. Over time, a common understanding of terms and concepts begin to emerge as academics and practitioners work together to tackle issues.

To address the common notion that genomics is a separate field, rather than an area that is becoming increasingly relevant to almost every disease and public health program area, we have learned the value of framing genomics as an additional tool for informing and addressing public health issues. For example, we conducted a project to examine the impact of genomics on public health efforts to reduce asthma morbidity and mortality by using a consultative process that engaged public health professionals, researchers, health care providers, and community representatives in dialogue about this issue. The final conclusions and recommendations drawn from this process are summarized in a final report, *Asthma Genomics: Implications for Public Health* (15). We hope that by engaging a variety of experts in the examination and dialogue process and by broadly disseminating the final report, those involved in asthma public health efforts at the local, state, and national levels will be more prepared to manage issues that may arise with the use of genomics to prevent, diagnose, and treat asthma and will think more about how genomics might play a role in reducing the effects of this common disease.

Family history, which reflects the consequences of genetic susceptibilities, shared environment, and common behaviors, is a risk factor for almost all chronic diseases (7) and can be incorporated into efforts to address many diseases of public health importance. Although family history has been long collected within the medical setting, there have been few public health efforts promoting the use of family history as a tool for disease prevention (16). In 2003, the CDC funded three sites to answer many important questions regarding the use of family history in public heath and preventive medicine (17). In the meantime, however, state health agencies appear interested in playing a role in answering important questions, including the following: Can a simple family history tool accurately and reliably collect information from family members? Can disease information about an individual's relatives be used to inform their risk for disease? If so, would individuals found to be at increased risk be more likely to adopt lifestyle changes and participate in early detection and prevention strategies (16)?

Family history was a topic for which we identified opportunities for collaboration with the Washington State Diabetes Prevention and Control Program (DPCP). For example, we took part in an assessment of the Washington State Diabetes Public Health System Performance, which was aimed at identifying the strengths and weaknesses of the statewide diabetes public health program. Opportunities for improvement resulting from the assessment included capturing the nonidentified diabetics in the state, developing a robust research agenda relevant to diabetes public health practice, and developing strategies to work more closely with academic partners. To help the WA DOH address these identified gaps, we proposed to develop a research project involving the use of family history as a public health tool. The Washington State Collaborative (WSC) Adult Preventive Services, a systematic approach to health care quality improvement in which organizations and providers test procedural innovations and then share their experiences to accelerate learning and promote widespread implementation of best practices, was an ideal setting in which to perform this project. We plan to collaborate with DPCP to collect more structured family histories as part of the WSC's quality improvement efforts, as well as to provide training to physicians regarding the utility of family history information.

After solid relationships with public health practitioners have been developed, in which practitioners begin to understand how genomics can be used as a tool for addressing issues of public health importance and academics begin to understand how genomics fits within the context of current public health programs and priorities, collaborative projects aimed at integrating genomics into public health are more easily identified. Funding for collaborative projects is an important issue, as state health departments often do not have the resources to carry out special projects.

In some instances, genomics-related projects can be proposed within the context of larger public health program proposals. For example, as a result of our efforts to demonstrate the relevance of family history to diabetes public health efforts, we were invited by the WA DOH to present information on the use of family history as a potential public health tool to those communities submitting proposals to the U.S. Department of Health and Human Services initiative, *Steps to a HealthierUS*. We suggested several potential community projects, including a community campaign to increase knowledge of family history. By incorporating family history into a comprehensive disease prevention and health promotion strategy, the communities would address many objectives of *Steps to a HealthierUS*. The Washington communities received funding, and we plan to assist them in identifying ways in which family history can be used to meet their goals.

Proposals also can be developed around other projects. For example, to evaluate the potential use of family history information, we are in the process of identifying funding to pilot a family history tool in a sample of clinics participating in the WSC. In addition, some projects can be best implemented in conjunction with the four states that have obtained funding to address genomics and chronic disease (14). For example, we are working with the health departments of the four states in reviewing existing family history questions included on various state surveys (e.g., Behavioral Risk Factor Surveillance System) and in developing new questions about family history for such surveys. Lastly, we have had discussions with OHD about collaborating on the development of a genomics awareness campaign for the health agency.

## Benefits of Using Academic–Practice Collaborations

Because they have only been in existence for two and a half years, it is difficult to fully assess the benefits of the collaborations the centers have developed with their state public health agency partners. Several benefits of these academic–practice partnerships, however, have been casually observed. Academics have gained an awareness of what public health practice means, including an appreciation for the valuable expertise held by program staff and the day-to-day work they carry out. As a result, many have become involved in practice-based research, teaching, and service activities that they would have been less likely to consider prior to the exposure to public health practice afforded to them through these centers.

State public health departments are also taking better advantage of the expertise held by public health genomics researchers. For example, requests to the centers for guest speakers at conferences and representatives for statewide committees and taskforces have increased. Both academics and practitioners have come together to identify collaborative projects and opportunities to participate in research aimed at questions of public health importance.

Lastly, public health students have gained invaluable experience with real-world public health genomics issues through their involvement in projects carried out through the centers, creating a new generation of public health professionals with exposure to genomics in practice. In some cases, public health departments have benefited because these students have tackled valuable projects that they otherwise would not have had resources to address.

The academic–practice partnerships created through the centers for Genomics and Public Health are slowly transforming the landscape of public health genomics research and practice. The well-traveled bridge between the centers and state health departments has created partnerships in which both sides benefit and flourish. The work of academics is informed and enriched by real-world issues that affect real populations while the work of public health practitioners is sharpened with the developing knowledge and new approaches the academy has to offer. This burgeoning synergy, strengthened by time, shared experiences, and successes, is ultimately greater than the sum of its parts and will be an important element for the genomics revolution to maximally benefit public health.
